# Dermatofibrosarcoma protuberans misdiagnosed as lipoma: a case report and literature review

**DOI:** 10.3389/fonc.2026.1697836

**Published:** 2026-03-12

**Authors:** Xinghao Yang, Feiyu Yin, Haiyang Jiang, Yuqin Wang, Longguo Dai, Zhishun Yang, Xusong Pang, Chongjian Zhang

**Affiliations:** 1 Department of Gastrointestinal Surgery, The Second Affiliated Hospital of Kunming Medical University, Kunming Medical University, Kunming, Yunnan, China; 2 Department of Urology, Peking University Cancer Hospital Yunnan, Yunnan Cancer Hospital, The Third Affiliated Hospital of Kunming Medical University, Kunming Medical University, Kunming, Yunnan, China

**Keywords:** case report, dermatofibrosarcoma protuberans, diagnosis, lipoma, surgery

## Abstract

Dermatofibrosarcoma protuberans (DFSP) is a relatively rare locally aggressive skin tumor. In its early stages, it can be easily confused with benign tumors such as lipomas and fibromas. Negative surgical margins represent the preferred treatment method for DFSP. For patients with advanced, unresectable disease, imatinib is a good option. The high rate of local recurrence remains a significant issue affecting patient prognosis. Here, we report on a case of a 30-year-old male patient with DFSP originating from the pubic symphysis. Due to the nonspecific clinical signs and the imaging findings, coupled with the rarity of DFSP and its location, the patient was misdiagnosed as having a lipoma and underwent surgical treatment. It was only through pathological examination that a diagnosis of DFSP was confirmed. This case emphasizes the importance of considering DFSP in the differential diagnosis of soft tissue masses, even in atypical locations.

## 1 Background

Dermatofibrosarcoma protuberans (DFSP) is a relatively rare, locally aggressive skin tumor (1, 2). Prior to its transformation into fibrosarcoma, the risk of metastasis for DFSP is low (3, 4). The trunk and proximal limbs are the most common sites for DFSP occurrence (5). However, due to its nonspecific imaging characteristics, DFSP can easily be confused with other conditions such as lipomas. According to the National Comprehensive Cancer Network (NCCN) guidelines, enhanced MRI is the preferred diagnostic method for assessing tissue involvement and depth in DFSP, although the final diagnosis is based on pathological examination (1, 4). Negative surgical margins represent the preferred treatment for DFSP, and Mohs micrographic surgery (MMS) is also utilized as a real-time margin assessment method for DFSP treatment. For patients with unresectable, recurrent, and/or metastatic DFSP, imatinib has been approved by the US Food and Drug Administration (FDA) for treatment. While the overall survival rate for DFSP is good, its high recurrence risk remains a potential concern, making regular follow-up extremely important (4).

We report on a case of a protruding DFSP originating from the pubic symphysis. Due to the rarity of both the disease and its location, we initially misdiagnosed it as a lipoma in front of the pubis, which led to failure to achieve accurate surgical margins. Therefore, we share the uniqueness of this case to provide a reference for clinicians around the world.

## 2 Case report

### 2.1 Patient information and clinical findings

**Figure 1:**
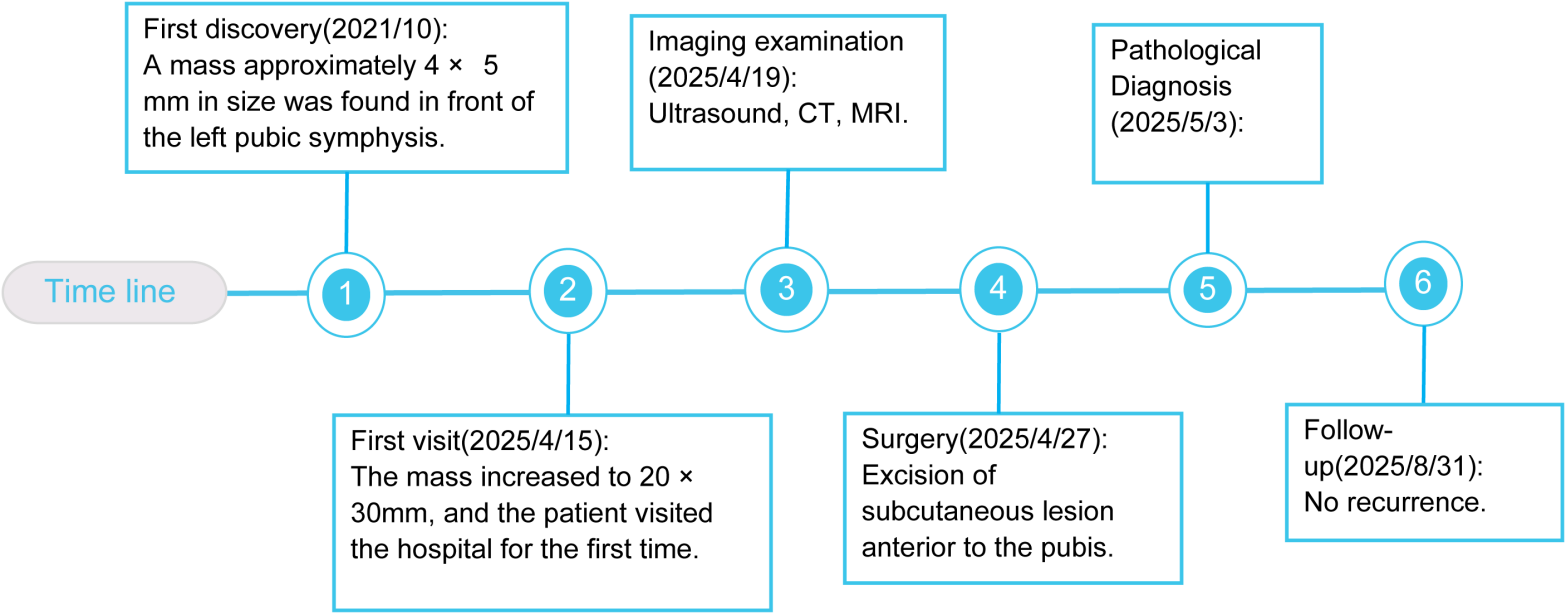
Timeline with relevant data from the patient’s history. *CT*, computed tomography; *MRI*, magnetic resonance imaging.

A 30-year-old male Chinese patient presented with a subcutaneous mass in the pubic region that had been present for 4 years. In 2021, the patient inadvertently discovered a soft mass approximately 4 mm × 5 mm in size with clear boundaries in front of the pubic symphysis. By April 2025, the mass had hardened and enlarged without any treatment. Physical examination revealed three fused hard masses in the suprapubic area, measuring 20 mm × 30 mm, with slight erythema of the overlying skin. The rest of the examination showed no particular abnormalities. The patient has no previous history of any special illness. There are no unusual medical conditions or cancers in the family (**Figure 1**).

### 2.2 Diagnostic assessment

**Figure 2:**
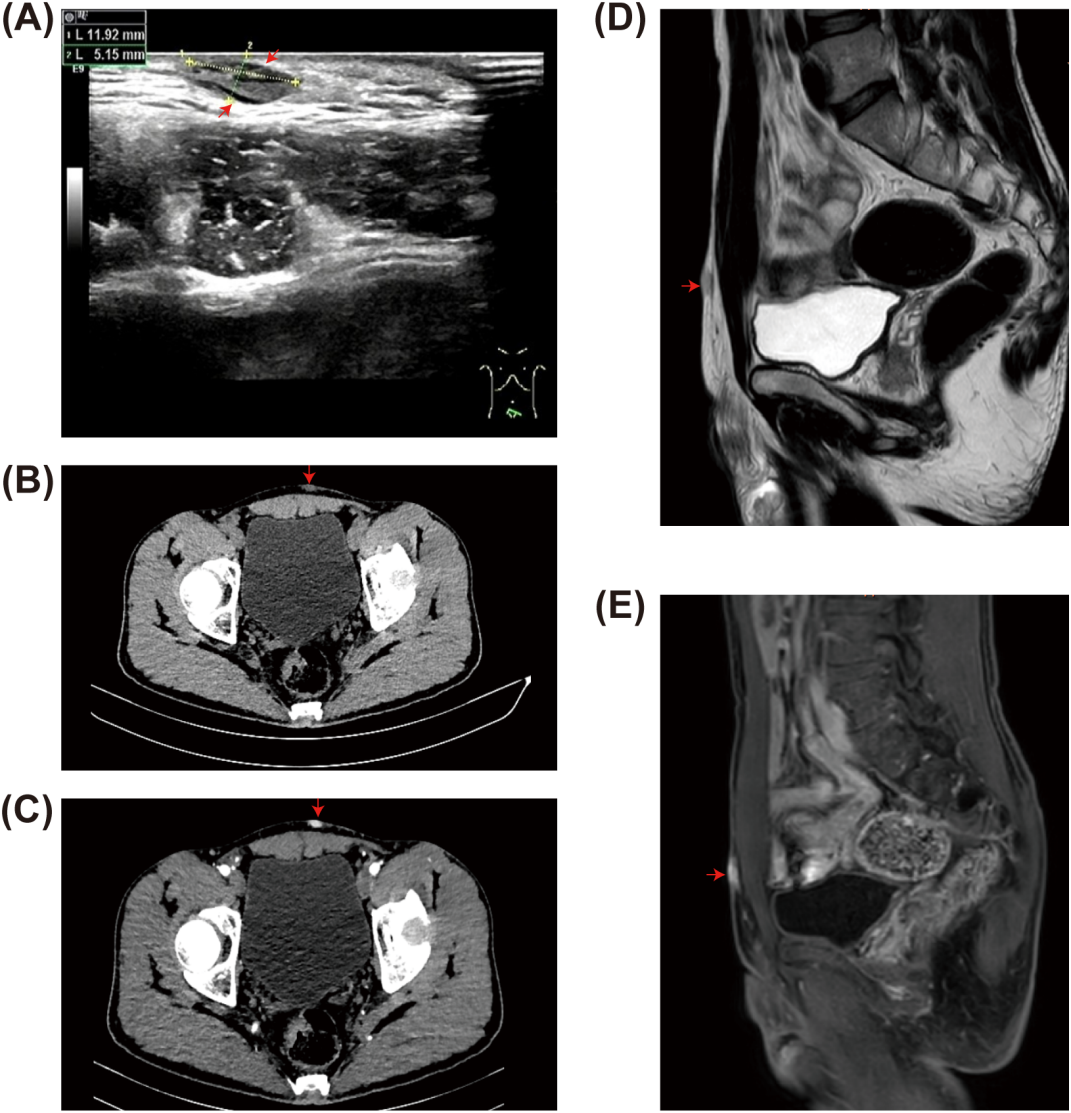
Imaging features of dermatofibrosarcoma protuberans (DFSP). Ultrasound (US) image illustrating the echotexture and morphological features of the tumor. **(A)** The *two lines* indicate the two diameter lines of the largest mass measured under US. **(B, C)** Computed tomography (CT) scans showing the extent and enhancement pattern of the mass. **(D, E)** Magnetic resonance imaging (MRI) demonstrating the lesion with characteristic signal patterns.

In terms of imaging examinations, we initially selected the simplest ultrasound (US). This revealed two to three well-defined, irregularly shaped hypoechoic areas within the skin layer and subcutaneous soft tissue of the pelvic region, suggesting benignity (**Figure 2A**). To prioritize the assessment of characteristic calcifications and clarify their adjacency to bony structures, we opted for a computed tomography (CT) (**Figures 2B, C**). Simultaneously, we added an abdominal magnetic resonance imaging (MRI) as a supplementary examination to determine the true extent of tumor infiltration in the soft tissue and its relationship with surrounding structures (**Figures 2D, E**). Two examinations revealed multiple nodular thickening of the skin in the left anterior pelvic wall, merging with one another (approximately 33 mm × 19 mm). MRI further demonstrated restricted diffusion (high signal on diffusion-weighted imaging, DWI). Although the lesion demonstrated heterogeneous enhancement, its overall imaging appearance, including well-defined borders and lack of aggressive infiltration, still favored a benign process.

In summary, despite the presence of atypical features such as infiltrative growth and restricted diffusion, the overall imaging appearance, including well-defined borders and the absence of aggressive infiltration, still suggests a benign process, and there was a lack of evidence indicating malignancy. Therefore, preoperatively, atypical anterior pelvic lipoma remains the primary consideration.

### 2.3 Therapeutic intervention

**Figure 3:**
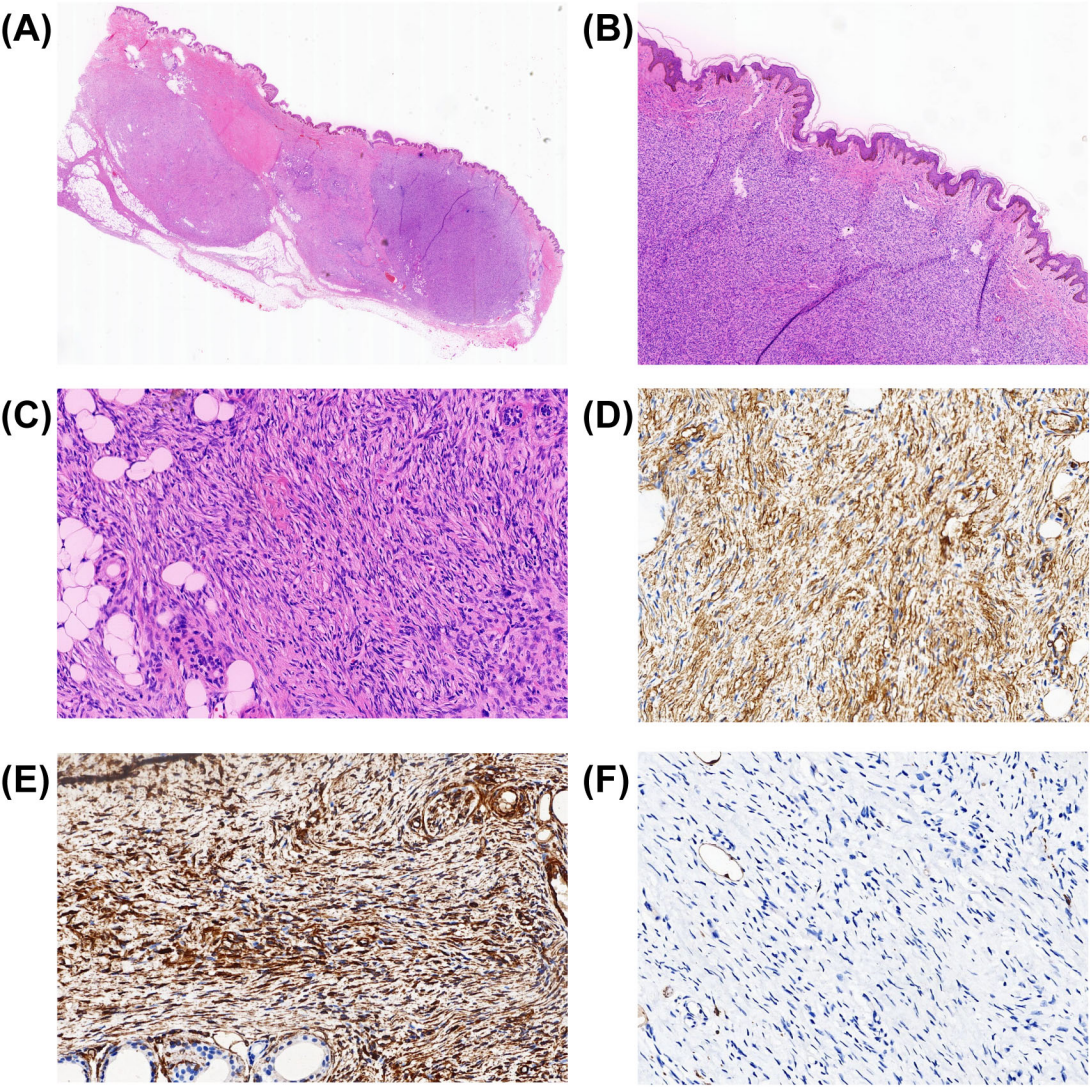
Histologic appearance of dermatofibrosarcoma protuberans (DFSP). **(A–C)** Hematoxylin–eosin (HE) staining. Microscopic examination of the skin mass in front of the pubic symphysis reveals spindle-shaped cells arranged in a characteristic storiform pattern. The infiltration of adipose tissue in a honeycomb pattern is indeed observed and is characteristic of DFSP. The images correspond to the panoramic view of the section **(A)** and magnifications of ×40 **(B)** and ×200 **(C–F)** Immunohistochemistry (IHC) staining. Strong CD34 expression in the tumor cells of DFSP, with the image corresponding to a magnification of ×200 **(D)**. Strong Vim expression in the tumor cells of DFSP, with the image corresponding to a magnification of ×200 **(E)**. Complete absence of S-100 in DFSP, with the image corresponding to a magnification of ×200 **(F)**.

We performed an excision of the subcutaneous lesion anterior to the pubic symphysis. Based on the preoperative imaging comprehensive assessment leaning toward a benign lesion (lipoma), during the procedure, we completely excised a mass measuring approximately 25 mm × 30 mm along the edge of the lesion by 10 mm. The texture of the mass was clearly delineated from the surrounding tissue. Inside, we identified a palpable, firm, well-defined round nodule measuring 15 mm × 10 mm. The intraoperative findings further supported the initial benign assessment. Under the microscope, we determined spindle cell lesions in the dermis (**Figures 3A–C**) and a strong positivity for CD34 and Vim (**Figures 3D, E**). On the other hand, S-100 (**Figure 3F**), desmin, ß-catenin, and STAT6 were negative. Therefore, a diagnosis of DFSP was made. After 1 week, the patient’s wound exhibited normal scabbing and gradual healing after the dressing change. Before discharge, the patient declined our recommendation for a second surgery to ensure adequate negative margins.

### 2.4 Follow-up

After 6 months, the patient was followed up via telephone. The patient reported that there were no palpable masses in the surgical area in front of the pubic symphysis, and no imaging studies had been conducted.

### 2.5 Patient perspective

Our patient has also expressed his experiences regarding this medical visit: “I believe that the diagnostic and therapeutic process was responsible, as the physician provided comprehensive information regarding all imaging findings and the surgical plan prior to the operation. Although the postoperative pathological results were not ideal, the hospital communicated openly and suggested a second surgery. However, I personally feel that the core issue has been resolved, and therefore, I do not accept the second surgery. Additionally, considering the distance of my residence, a second operation would delay my work and impose a financial burden. Moving forward, I will monitor for any signs of recurrence, and should any abnormalities arise, I will seek medical attention promptly.”

## 3 Discussion

The main highlight of this case report is the description of a deceptive rare skin tumor. DFSP is a relatively rare locally aggressive skin tumor that originates from the dermis of the skin, accounting for approximately 1% of all soft tissue tumors (1, 6). DFSP can occur in the trunk (42%–52.7%), lower limbs (12.3%–25.4%), head and neck (10.6%–17.5%), and upper limbs (6.8%–12.7%) (7). However, its occurrence in front of the pubic symphysis is uncommon. Through our search in the PubMed and FMRS databases, we did not find any cases occurring in front of the pubic symphysis, while there are only three English case reports in the adjacent lower abdominal area (8–10) (**Table 1**). The pathogenesis of DFSP is primarily associated with the translocation between chromosomes 17 and 22, specifically *t* (17, 22)(q22;q13), which results in the fusion of the *PDGFB* gene with the *COL1A1* gene. This fusion leads to the formation of the *COL1A1*–*PDGFβ* fusion gene, which serves as a significant molecular marker for DFSP (4). However, due to the error in our initial diagnosis, the corresponding molecular testing was not performed on the patient.

**Table 1:** Review of the literature on dermatofibrosarcoma protuberans (DFSP) of the lower abdomen.

Author	Gender	Age (years)	Tumor size	IHC	Primary surgery	Other treatments	Recurrence	Metastasis	CT	US	MRI
Hsieh et al.	Male	59	80 mm × 110 mm	Not performed	WLE + Skin flap grafting	Radiotherapy	4 years	Not performed	Not performed	Not performed	Not performed
Saeki et al.	Male	43	40 mm × 32 mm	CD34 (+) S-100 (−)	WLE	Not performed	No	Not performed	Not performed	Not performed	Not performed
Essid et al.	Male	45	Not performed	Not performed	WLE + Skin flap grafting	Not performed	No	Not performed	Subcutaneous fat infiltration into deep muscles	Not performed	Not performed

*IHC*, immunohistochemistry; *CT*, computed tomography; *US*, ultrasound; *MRI*, magnetic resonance imaging; *WLE*, wide local excision.

Initially, it presents as a hard mass, skin nodules, or plaques, fixed onto the skin surface and can be pushed (1). Over time, the tumor may exhibit infiltrative growth resembling pseudopodia into deeper tissues, invading subcutaneous tissue, muscle, and even bone. The surface of the lesion is prone to ulceration and may cause local compressive symptoms, which can be differentiated from epidermoid cysts that originate from hair follicles, are expressible, and are often presented due to inflammatory pain (11). However, due to the lack of specific clinical symptoms in the early stages of this disease, it can easily be confused with other benign tumors. Therefore, additional auxiliary examinations are necessary to assist in the diagnosis.

In our case, the patient was in the early stage of DFSP, with the lesion located superficially in front of the pubic symphysis. Clinically, a painless induration is palpated, and imaging also revealed a well-defined mass. These characteristics are similar to those of common benign tumors such as lipomas, which lack specific manifestations. In addition, DFSP is relatively rare in clinical practice, and both clinicians and radiologists have limited diagnostic experience with its early and atypical presentations. When faced with a well-defined superficial mass, the diagnostic thought process naturally leans toward more common benign lesions. This is also the primary reason for our misdiagnosis.

Therefore, rigorous imaging examinations are crucial for assessing the extent and characteristics of DFSP. Under US, DFSP typically presents as a well-defined, oval-shaped hypoechoic mass, which may exhibit lobulated features or a mixture of high and low echogenicity. Its key characteristic is aggressive growth, with specific patterns including: superficial invasion only, finger-like projections, deep echogenic zone invasion, and mixed infiltrative types (12, 13).

As the preferred imaging modality for soft tissue, MRI is the best choice (1). The NCCN guidelines indicate that enhanced MRI is the preferred diagnostic method for the assessment of tissue involvement and depth in DFSP (4). On T1-weighted (T1W) imaging, DFSP typically appears homogeneous. On T2-weighted (T2W) imaging, DFSP generally shows increased signal intensity. Recurrent DFSP typically presents as distinct nodules or mass images, with prolonged relaxation times on T1W and T2W. If there is increased intensity on T2W and enhancement on T1W imaging, nodules within the tumor capsule indicate a higher risk of recurrence. DFSP appears as well-defined nodular structures on CT, with a density similar to that of skeletal muscle (13).

The radiological differential diagnosis still faces significant challenges, primarily manifested in the overlap of imaging features. On one hand, DFSP shares similarities with certain benign lesions, such as desmoid-type fibromatosis originating from the fascia, dermatofibromas that often present as homogeneous solid nodules, and solitary nodular spindle cell lipomas, making differentiation difficult based solely on morphology. On the other hand, its characteristics, such as infiltrative growth patterns and heterogeneous T2W signals, exhibit considerable overlap with low-grade fibromyxoid sarcoma, myofibroblastic sarcoma, and other low-grade malignant sarcomas. This overlap results in difficulties in achieving accurate differentiation based solely on imaging, necessitating that the final diagnosis relies on pathological examination for clarification.

Pathology is the best diagnostic method for DFSP. It has multiple subtypes, with the classic type being the most common. However, the risk of local recurrence, metastasis, and mortality in fibrosarcomatous DFSP (FS-DFSP) is significantly higher than that in the classic type (14). The classic type of DFSP exhibits spindle cells arranged in a storiform pattern under the microscope. In the early stages of the disease, a “Grenz zone” can be observed, which is characterized by a narrow band of tumor-free cells between the lesion and the overlying epidermis (15, 16). When tumor cells infiltrate the subcutaneous fat, a lobular appearance may be observed. This characteristic aids in distinguishing it from spindle cell lipomas, which also contain mature adipocytes, but have a different structure. The classic type of DFSP is characterized by CD34 positivity and factor XIIIa negativity, which is the opposite of the Dermatofibroma (DF). Studies indicate that DFSP can exhibit positive results for apolipoprotein-D (Apo-D) membrane and weak positivity for epithelial membrane antigen (EMA), while H3K27me3 and GRIA2 can also show positive expression. These negative indicators are helpful in excluding S-100-positive lipomas, β-catenin-positive desmoid tumors, SMA- and desmin-positive leiomyomas, and low-grade myofibroblastic sarcomas, as well as STAT6-positive solitary fibrous tumors (1, 17).

In our case, when we aimed to confirm the diagnosis of lipoma through pathological examination, we received a negative result. Under the microscope, spindle-shaped cells were arranged in a storiform pattern, which is inconsistent with the mature adipocytes and morphologically similar spindle-shaped cells that constitute lipoma. In particular, S-100 was negative while CD34 was positive.

The standard treatment for DFSP is surgical excision of the lesion, which includes encompassing marginal excision, wide local excision (WLE), and MMS (18). It is recommended to excise with a margin of 2–3 cm to reduce the risk of local recurrence (1, 19). Despite the recommendation by WLE to maintain a margin of 2–4 cm, the local recurrence rate remains as high as 60% (20). MMS allows for the microscopic examination of continuous horizontal sections (18), resulting in a recurrence rate of approximately 1.5%. In our case, due to a misdiagnosis, a complete margin resection was not performed, which poses a certain risk of recurrence. This is also a limitation in our treatment approach. Of course, we respect the patient’s decision regarding the refusal of a second surgery. We advised the patient to have regular follow-ups and to seek timely surgical treatment in case of recurrence.

For unresectable DFSP, targeted therapy is a promising option. Imatinib inhibits the proliferation of DFSP by inhibiting the platelet-derived growth factor receptor tyrosine kinase. It has currently been approved by the FDA for unresectable, recurrent, or metastatic DFSP (21). In addition, radiotherapy can be utilized for patients who are not suitable for surgical treatment. Whether used alone or in conjunction with conservative surgical resection, radiotherapy can achieve lasting local disease control for DFSP (19), particularly in cases with positive surgical margins, where it can serve as a means to improve prognosis (1). For our patient, if subsequent imaging or clinical manifestations reveal a tendency for recurrence, genetic testing can be performed to clarify whether there are indications for targeted therapy. In addition, we may recommend that the patient undergo adjunctive radiotherapy.

## 4 Conclusion

DFSP is a rare locally invasive skin tumor primarily occurring on the trunk and limbs. Its early clinical manifestations tend to resemble those of benign soft tissue tumors, which often leads to the possibility of DFSP being overlooked during diagnosis, resulting in inaccurate treatment of the disease. This case highlights that DFSP may arise in atypical anatomical sites, such as the prepubic region, where it may closely mimic benign soft tissue lesions. Greater awareness of these unusual presentations is essential to avoid misdiagnosis and incomplete surgical excision. It is hoped that this will provide a reference for clinicians in the medical community.

## Data Availability

The original contributions presented in the study are included in the article/supplementary material. Further inquiries can be directed to the corresponding author.
